# Preparation of Polyethylene Glycol-Ginsenoside Rh1 and Rh2 Conjugates and Their Efficacy against Lung Cancer and Inflammation

**DOI:** 10.3390/molecules24234367

**Published:** 2019-11-29

**Authors:** Ramya Mathiyalagan, Chao Wang, Yeon Ju Kim, Verónica Castro-Aceituno, Sungeun Ahn, Sathiyamoorthy Subramaniyam, Shakina Yesmin Simu, Zuly Elizabeth Jiménez-Pérez, Deok Chun Yang, Seok-Kyu Jung

**Affiliations:** 1 Graduate School of Biotechnology, College of Life Science, Kyung Hee University, Yongin-si, Gyeonggi-do 17104, Korea; ramyabinfo@gmail.com (R.M.); simu.sha2@gmail.com (S.Y.S.); zejp78@gmail.com (Z.E.J.-P.); 2 Department of Oriental Medicinal Biotechnology, College of Life Science, Kyung Hee University, Yongin-si, Gyeonggi-do 17104, Korea; chaow@sdut.edu.cn (C.W.); yeonjukim@khu.ac.kr (Y.J.K.); ca.veronica@khu.ac.kr (V.C.-A.); se8688@gmail.com (S.A.); s.sathiyamurthi@gmail.com (S.S.); 3 Institute of Biomedical Research, School of Life Sciences, Shandong University of Technology, Zibo 255000, Shandong, China; 4 Department of Biotechnology, Dr.N.G.P., Arts and Science College, Coimbatore 641048, Tamil Nadu, India

**Keywords:** Korean ginseng, ginsenoside Rh1, ginsenoside Rh2, polyethylene glycol, conjugation, nanoparticles, lung cancer, inflammation

## Abstract

Low solubility and tumor-targeted delivery of ginsenosides to avoid off-target cytotoxicity are challenges for clinical trials. In the present study, we report on a methodology for the synthesis of polyethylene glycol (PEG)-ginsenoside conjugates through a hydrolysable ester bond using the hydrophilic polymer polyethylene glycol with the hydrophobic ginsenosides Rh1 and Rh2 to enhance water solubility and passive targeted delivery. The resulting conjugates were characterized by ^1^H nuclear magnetic resonance (^1^H NMR) and Fourier-transform infrared spectroscopy (FT-IR). ^1^H NMR revealed that the C-6 and C-3 sugar hydroxyl groups of Rh1 and Rh2 were esterified. The conjugates showed spherical shapes that were monitored by field-emission transmission electron microscopy (FE-TEM), and the average sizes of the particles were 62 ± 5.72 nm and 134 ± 8.75 nm for PEG-Rh1and PEG-Rh2, respectively (measured using a particle size analyzer). Owing to the hydrophilic enhancing properties of PEG, PEG-Rh1 and PEG-Rh2 solubility was greatly enhanced compared to Rh1 and Rh2 alone. The release rates of Rh1 and Rh2 were increased in lower pH conditions (pH 5.0), that for pathophysiological sites as well as for intracellular endosomes and lysosomes, compared to normal-cell pH conditions (pH 7.4). In vitro cytotoxicity assays showed that the PEG-Rh1conjugates had greater anticancer activity in a human non-small cell lung cancer cell line (A549) compared to Rh1 alone, whereas PEG-Rh2 showed lower cytotoxicity in lung cancer cells. On the other hand, both PEG-Rh1 and PEG-Rh2 showed non-cytotoxicity in a nondiseased murine macrophage cell line (RAW 264.7) compared to free Rh1 and Rh2, but PEG-Rh2 exhibited increased efficacy against inflammation by greatly inhibiting nitric oxide production. Thus, the overall conclusion of our study is that PEG conjugation promotes the properties of Rh1 for anticancer and Rh2 for inflammation treatments. Depends on the disease models, they could be potential drug candidates for further studies.

## 1. Introduction

According to a World Health Organization report (https://www.who.int/news-room/fact-sheets/detail/cancer), lung cancer (1.76 million deaths) is the leading cause of cancer mortality in humans [1], beating out colorectal (862,000 deaths), stomach (783,000 deaths), liver (782,000 deaths), and breast (627,000 deaths) cancer. Chemotherapy is an important part of cancer treatment, and numerous anticancer drugs have been reported [2]. However, pitfalls exist in the physiochemical properties of cancer drugs, including poor aqueous solubility, a short half-life in the body, low bioavailability, and target-specific cytotoxicity in the cancer microenvironment [3,4]. These factors lead to poor antitumor effects, systemic toxicity, and other side effects in patients and reduce the quality of life and clinical applications. To surmount these issues, numerous water-soluble polymers with good biocompatible and biodegradable properties have been applied as carriers for the delivery of these anticancer drugs, and they have exhibited various advantages [5,6,7,8,9]. Among these, polyethylene glycol (PEG) is a water-soluble, nontoxic polymer that has been approved by the Food and Drug Administration (FDA) as “Generally Regarded as Safe (GRAS)”, and it is used in targeted delivery [10,11,12]. There are some PEGylated drugs that have been approved by the FDA, as well as four small-molecule drugs in clinical trials [13]. PEG has been reported to improve the solubility of drugs [14], increase the circulation time of drugs in the bloodstream by preventing them from reticuloendothelial clearance, enhance accumulation in tumor tissues through an enhanced permeation and retention (EPR) effect, induce a stimuli-responsive release of the conjugated drugs into the tumor microenvironment by reducing the cytotoxicity in the normal tissues, improve the half-life of drugs in the body compared to free drugs, and prevent the degradation of drugs by intestinal enzymes [13,15,16,17,18,19,20,21,22,23].

Ginsenosides are versatile phytochemical drug candidates from the oriental medicinal herb *Panax ginseng* that have been reported on for their various pharmacological efficacies [24]. Various technologies have been applied to elucidate the biosynthesis mechanism of ginsenosides in ginseng and its bioconversion of major to minor ginsenosides (which possess more numerous bioactivities than the major ginsenosides) [25,26,27]. Structurally, ginsenosides are classified into protopanaxadiols (PPDs), protopanaxatriols (PPTs), and oleanane-type saponins according to the glycosidic linkage of sugar chains to their triterpenoid aglycone [28]. Further, ginsenosides are grouped as major and minor based on the number of sugar molecules attached [28]. Although various efficacies of ginseng crude extract and ginsenosides have been reported, the majority of major ginsenosides are hydrolyzed into minor ginsenosides after oral administration [29]. Besides improved pharmacological efficacies and the better systemic circulation of minor ginsenosides (PPD type CK (Compound K), Rh2, and PPD aglycone and PPT type Rh1, F1, and PPT aglycone), their hydrophobicity, targeted delivery into pathophysiological sites such as tumors, and propensity to cause inflammation by avoiding normal cell cytotoxicity, are crucial factors in applying ginsenosides in clinical trials [24]. Moreover, conjugates, especially those with nanoparticles, have gained more attention than the original form of ginsenosides due to their enhanced pharmacological effects [17,30,31,32,33,34]. In recent years, studies on ginsenoside Rh1 (PPT type) [35,36,37,38,39,40,41,42] and Rh2 (PPD type) [43,44,45,46,47,48,49] have focused on their anti-inflammatory, anticancer, and immune conditioning abilities. Rh2 and Rh1 both have a similar structure, but vary in terms of one glucose molecule at C-3 (PPD type) and C-6 (PPT type): they also have higher cell uptake ratios compared to major ginsenosides. Moreover, new standardization practices are preferred for ginseng products with minor ginsenosides, such as Rh2. Accordingly, in the present study, PEG conjugation was applied to ginsenosides Rh1 and Rh2 to increase their solubility, their cytotoxicity in tumor cells, and their anti-inflammatory properties, with lower cytotoxicity in normal cells. The resultant PEG-Rh1and PEG-Rh2 conjugates were characterized using standard techniques (^1^H NMR, FT-IR, and field-emission transmission electron microscopy (FE-TEM)) and particle size analysis. Solubility and the in vitro release of Rh1 and Rh2 were studied under pathophysiological (pH 5.0) and physiological (pH 7.4) conditions. In addition, in vitro cytotoxicity was assessed in a human lung cancer cell line (A549) and in murine macrophage cell line (RAW 264.7). Finally, the anti-inflammatory properties of PEG-Rh1 and PEG-Rh2 were evaluated through the inhibition of nitric oxide (NO) production in the RAW 264.7 cell line.

## 2. Results and Discussion

We aimed to increase the solubility, antitumor activity, and anti-inflammation activity of Rh1 and Rh2 and to decrease cytotoxicity to normal cells. Self-assembled PEG micelles were prepared with an acid-liable ester bond. The conjugates were expected to accumulate more in the tumor tissues through an EPR effect as well as through inflammation and to release Rh1 and Rh2 in the intracellular lysosome and endosome due to acidic pH conditions. Further, Rh1 and Rh2 were expected to target the nucleus to degrade cancer cell genetic materials, as is illustrated in the graphical abstract.

### 2.1. Synthesis and Physiochemical Characterization of PEG-Rh1 and PEG-Rh2 Conjugates

**Figure 1:**
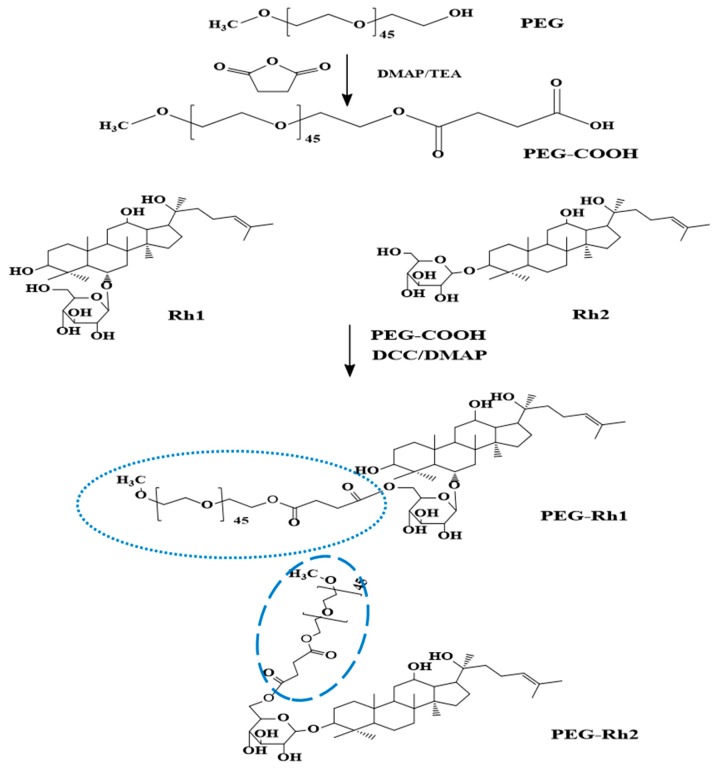
Schematic illustrations of polyethylene glycol (PEG)-Rh1 and PEG-Rh2 conjugate syntheses.

**Figure 2:**
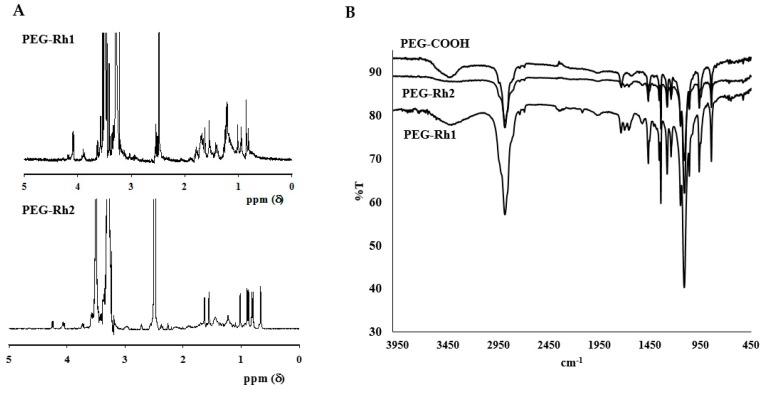
^1^H NMR (**A**) and FT-IR (**B**) spectra of the PEG-Rh1and PEG-Rh2 conjugates.

Initially, the chemical synthesis of the PEG-ginsenoside conjugates was completed in two steps, as shown in Figure 1. In the first step, the hydroxyl group in PEG was modified to a carboxyl group (PEG-COOH), which was confirmed by ^1^H NMR to have the characteristics of a succinic acid peak at 2.6 ppm. Next, the ester bonds between PEG-COOH and Rh1 and Rh2 were synthesized and the ester bond peaks were confirmed by ^1^H NMR (Figure 2A), as explained in Reference [18]. Though Rh1 and Rh2 ginsenosides possess similar structures and they differ only in terms of the glucose linkage at C-3 and C-6, the conjugation efficacy (228.5 μg of Rh1/1 mg PEG-Rh1 and 192 μg of Rh2/1 mg PEG-Rh2) greatly varied (more so than with other reported conjugates) [18].

**Figure 3:**
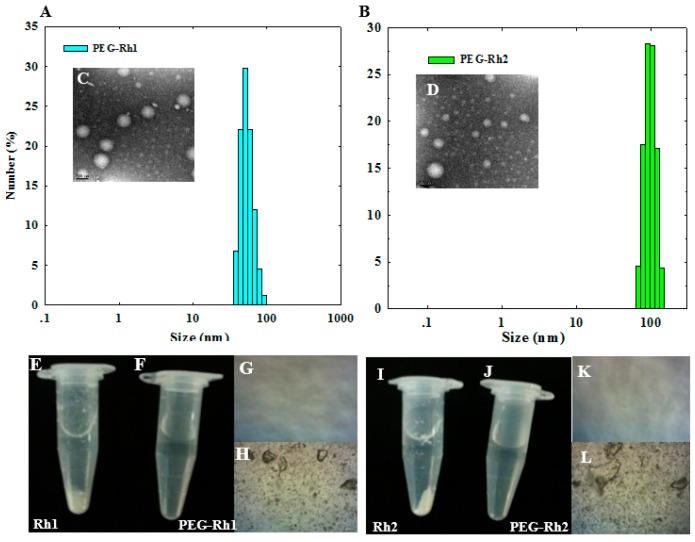
Particle size and spherical morphology of the PEG-Rh1 (**A**,**C**) and PEG-Rh2 (**B**,**D**) conjugates in aqueous solution. The scale bar in (**C**,**D**) represents 200 nm. Non-soluble precipitates of free Rh1 (**E**,**H**) and Rh2 (**I**,**L**) compared to the increased solubility of the PEG-Rh1 (**F**,**G**) and PEG-Rh2 (**J**,**K**) conjugates.

Though Rh2 is a PPD-type ginsenoside, a single conjugated PEG molecule is similar to PEG-PPD [17] but is less than PEG-CK, in which two molecules of PEG are conjugated [18]. The ester bond between PEG-COOH and the ginsenosides (Rh1 and Rh2) was confirmed with FT-IR spectroscopy (Figure 2B), which correlated with previous reports [17,18]. In vivo antitumor studies have reported that low-molecular-weight methoxy poly(ethylene glycol) methyl ether (mPEG 2000) (a conjugate of the anticancer drug gambogic acid with an ester bond) shows enhanced antitumor effects [14]. The results from a particle size analyzer confirmed that the particle sizes of synthesized PEG-Rh1 and PEG-Rh2 in terms of their average diameters in aqueous solution were 62 ± 5.72 nm and 134 ± 8.75 nm, respectively (Figure 3A,B), which was smaller than the PEG-PPD conjugate (189 ± 15.69 nm) [17]: further, the morphology of the conjugates was spherical, as could be seen after FE-TEM (Figure 3C,D).

### 2.2. Solubility of the PEG-Rh1 and PEG-Rh2 Conjugates

PEG conjugation considerably enhances the solubility of several anticancer drugs, which further enhances their bioavailability and antitumor activities [16,22,23]. Due to the hydrophilic nature of PEG, it forms a hydrophilic outer layer by holding the hydrophobic ginsenosides Rh1 and Rh2, which form self-assembled micelles in aqueous medium. Thus, the prepared PEG-Rh1 and PEG-Rh2 conjugates were soluble in phosphate-buffered saline (PBS pH 7.4) or water at 2 mg PEG-Rh1/mL (equivalent weight of 10.4 mg/mL Rh1) and 2 mg PEG-Rh2/mL (equivalent weight of 8.7 mg/mL Rh1), whereas free Rh1 and Rh2 were insoluble at much lower concentrations (Figure 3). Increased solubility and anticancer activities in various hydrophobic anticancer drugs due to PEGylation have been reported [13,14,16,19,22,23].

### 2.3. pH-Dependent Release of Rh1 and Rh2 from the PEG-Rh1 and PEG-Rh2 Conjugates

**Figure 4:**
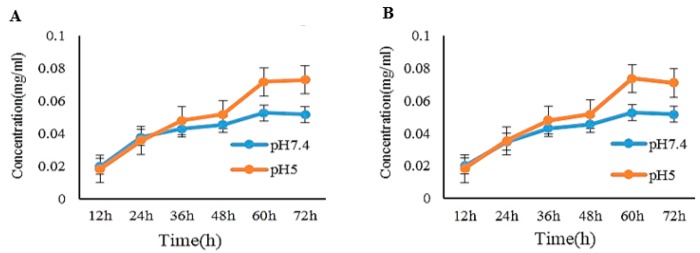
pH-dependent release of Rh1 and Rh2 from the PEG-Rh1 (**A**) and PEG-Rh2 (**B**) conjugates.

Polymer conjugates are able to reach tumor because of the tumor’s leaky vascular system (graphical abstract). Drugs are released into the tumor tissues through exposure to extra and intracellular stimuli. Specifically, pH-responsive drug conjugates have gained attention due to the variations in pH between tumor tissues and normal tissues. Since the intracellular tumor cell pH (pH 5.0–6.0) is lower than normal tissue pH conditions (pH 7.4), researchers have been able to develop pH-responsive prodrug formulations to improve activities [16,30]. The hydrolysis of PEG-Rh1 and PEG-Rh2 was monitored by incubating samples under different pH conditions (pH 5.0, pH 7.4) over different time points. The amounts of hydrolyzed Rh1 and Rh2 were determined by HPLC (Figure 4).

### 2.4. In Vitro Cytotoxicity Inhibition of lipopolysaccharide (LPS)-Induced nitric oxide (NO) Production by PEG-Rh1 and PEG-Rh2 Conjugates

**Figure 5:**
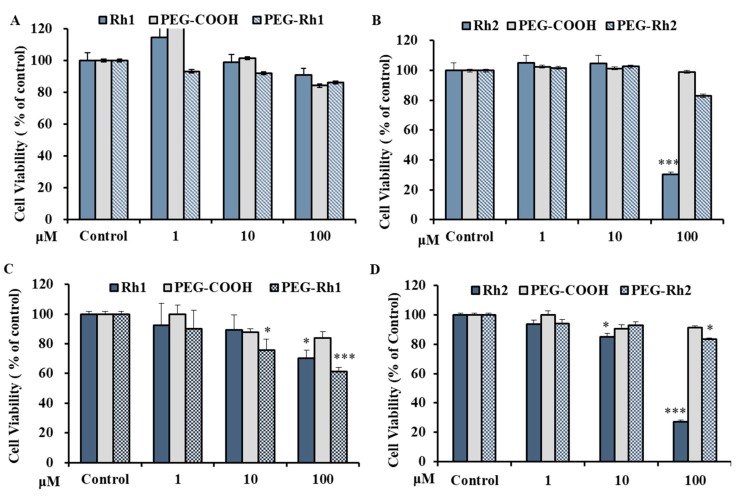
Cell cytotoxicity of PEG-Rh1 and PEG-Rh2 conjugates in RAW 264.7 cells (**A**,**B**) and A549 lung cancer cells (**C**,**D**) compared to the controls. All results are representative of three independent experiments; * *p* < 0.1 and *** *p* < 0.001 versus control group.

**Figure 6:**
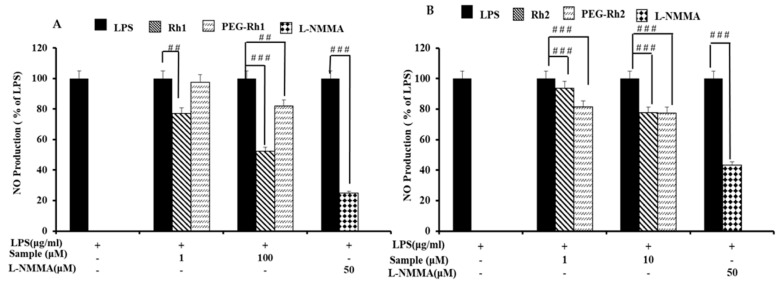
Effects of the PEG-Rh1 (**A**) and PEG-Rh2 (**B**) conjugates on nitric oxide production. LPS-treated RAW cells (to induce NO production and cause inflammation). LPS-lipopolysaccharide; NO-nitric oxide. ^##^ *p* < 0.01, ^###^ *p* < 0.001 as compared to the group treated with LPS alone.

Before evaluating the anti-inflammatory effects of PEG-COOH, ginsenoside Rh1, ginsenoside Rh2, PEG-Rh1, and PEG-Rh2, we examined their effects on the viability of RAW 264.7 cells. As is shown in Figure 5A,B, the viability of RAW 264.7 cells following treatment was not significantly decreased than control group. Since these ginsenosides showed no cytotoxicity effect on RAW 264.7 cells, we used up to 10 μM Rh2 and PEG-Rh2 or 100 μM Rh1 and PEG-Rh1 for the rest of our studies. As it has been previously reported that macrophage-like RAW 264.7 cells describe the actions of numerous anti-inflammatory mechanisms at the molecular stage [50], to further investigate whether PEG-Rh1 and PEG-Rh2 could function as inhibitors of nitric oxide (NO) production in this model, RAW 264.7 cells were stimulated with LPS (1 μg/mL) with or without cotreatment with ginsenoside Rh1, ginsenoside Rh2, PEG-Rh1, and PEG-Rh2. As is shown in Figure 6, ginsenoside Rh1, ginsenoside Rh2, PEG-Rh1, and PEG-Rh2 all dose-dependently repressed the NO production induced by LPS. Fascinatingly, it was observed that 10 μM PEG-Rh2 inhibited LPS-induced NO production in RAW 264.7 cells at a greater level compared to the 100 μM PEG-Rh1.

### 2.5. In Vitro Cytotoxicity of PEG-Rh1 and PEG-Rh2 in Lung Cancer A549 Cell Line

First, we evaluated the toxicity profile of PEG-COOH in A549 cells. Our results showed that, following the treatment of cells at concentrations up to 100 µM over a period of 48 h, cell viability was not significantly reduced. The efficacy of PEG-Rh1 was compared to free ginsenoside Rh1 (Rh1) in A549 cells at different concentrations for 48 h. We found that PEG-Rh1 exhibited high cytotoxicity compared to Rh1 at 100 µM (Figure 5C). In addition, we found that in A549 cells, PEG-Rh2 was less toxic than Rh2 up to 100 µM at 48 h (Figure 5D). Previously, it had been reported that PEGylated CK shows lower toxicity than its parent drug due to the slow release of drugs in pathophysiological pH condition [17,18]. However, the contributions of PEGylation to enhanced anticancer activities, bioavailability, solubility, and the targeted delivery of various anticancer drugs in vivo have been reported [4,16,20,23]. Thus, we suggest that the differences in responses between Rh1 and Rh2 after conjugation with PEG-COOH are related to differences in the glucose linkage at C-6 and C-3 in Rh1 and Rh2, respectively; the slow release of the compounds; and the high toxicity profile of free Rh2 in A549 cells. However, further experiments are needed to fully understand the differences between the molecular mechanism of PEG-Rh1 and PEG-Rh2.

## 3. Materials and Methods

### 3.1. Materials

Polyethylene glycol monomethyl ether (mPEG, Mn 2000 g/mol), *N*,*N*’-dicyclohexyl carbodiimide (DCC), dimethyl amino pyridine (DMAP), triethylamine (TEA), anhydrous 1,4-dioxane, and succinic anhydride were purchased from Sigma Aldrich Co. (St. Louis, MO, USA). The ginsenosides Rh1 and Rh2 were purchased from the Lab of Hanbangbio, Kyung Hee University, Yongin, South Korea. All other chemicals were of analytical grade and were used as received.

### 3.2. Synthesis of PEG-Rh1and PEG-Rh2Conjugates

PEG-Rh1 and PEG-Rh2 were synthesized as reported in Ramya et al. [18] with a few modifications, as shown in Figure 1. First, *α*-carboxy-ω-methoxy polyethylene glycol (mPEG-COOH) was synthesized, followed by conjugation with Rh1 or Rh2. Carboxylated PEG was prepared using succinic anhydride as follows: mPEG (Mw 2000 da) (1 g, 0.5 mmol), succinic anhydride (0.06 g, 0.6 mmol), DMAP (0.061 g, 0.5 mmol), and TEA (0.05 g, 0.5 mmol) were dissolved in anhydrous dioxane (10 mL) and stirred at room temperature for 24 h. PE-COOH was then precipitated in diethyl ether and further filtered and dried under vacuum. Next, mPEG-COOH (0.044 g), DCC (0.06 g), and DMAP were added to the stirred solution. After 15 min, Rh1 or Rh2 (dissolved in DMF) was added and stirred overnight, and the solution was further dialyzed (dialysis membrane (Mw cut-off: 3500)) against distilled water for 24 h. Finally, the dialysate was filtered (0.45-µM filter syringe) and lyophilized to obtain the PEG-Rh1 and PEG-Rh2 conjugates.

### 3.3. Characterizations of the Structure of the PEG-Rh1 and PEG-Rh2 Conjugates

The PEG-Rh1 and PEG-Rh2 conjugates were characterized by ^1^H NMR and FT-IR. For ^1^H NMR, samples were dissolved in deuterated dimethylsulfoxide (DMSO-*d*_6_), and the spectra were obtained at 300 MHz (JEOL, Tokyo, Japan). FT-IR spectra of the PEG-Rh1 and PEG-Rh2 conjugates were obtained using a Perkin-Elmer FT-IR spectrophotometer with KBr pellets. FE-TEM was used to observe the morphology of conjugates (JEM-2000F, JEOL, Tokyo, Japan) at 200 kV. To prepare TEM samples, a drop of sample solution was placed onto a 200-mesh copper grid and air-dried, and a drop of phosphotungstic acid (PTA) solution was then added. This was used for negative staining. The stability and size of the conjugates were obtained by a particle analyzer, and the samples were dissolved in phosphate-buffered saline (PBS, pH 7.4).

### 3.4. In Vitro pH-Dependent Release of Rh1 and Rh2 from the PEG-Rh1 and PEG-Rh2 Conjugates

The PEG-Rh1 and PEG-Rh2 conjugates were dissolved in pH 7.4 (PBS) buffer, transferred to a cellulose dialysis membrane (Molecular weight cut-off (MWCO: 3500), and then placed in 30 mL of PBS (pH 7.4) and acetate buffer (pH 5.0). The samples were moderately shaken at 37 °C and 120 rpm. At different time intervals, 5-mL samples were withdrawn and replaced with fresh medium. To calculate the quantity of released Rh1 and Rh2, the withdrawn samples were extracted three times with water-saturated *n-*BuOH, evaporated, further dissolved in HPLC-grade MeOH, and analyzed by HPLC (Agilent 1260, Palo Alto, CA, USA, C_18_ column, 3.0 × 50 mm, particle size 2.7 μm) with acetonitrile (solvent A) and distilled water (solvent B). The flow rate was 0.6 mL per min, and the detection wavelength was 203 nm.

### 3.5. Cell Culture

RAW 264.7 (murine macrophage obtained from the Korean Cell Bank (Seoul, South Korea)) cells were cultured at a density of 5 × 10^3^ cells/well in a 96-well microplate in RPMI-1640 medium containing 10% (v/v) fetal bovine serum (FBS) and 1% (v/v) penicillin/streptomycin. 

The human non-small cell lung cancer cell line (A549) was obtained from the Korean Cell Bank (Seoul, South Korea). The cells were cultured in a 37 °C humidified incubator in a 5% CO_2_ atmosphere and maintained in RPMI-1640 culture media (GenDEPOT, Inc., Barker, TX, USA) supplemented with 10% fetal bovine serum (FBS), 100 IU/mL penicillin, and 100 µg/mL streptomycin (Gibco-BRL, Gaithersburg, MD, USA).

### 3.6. Cell Viability Assay

The cytotoxicity of PEG-COOH, ginsenoside Rh1, ginsenoside Rh2, PEG-Rh1, and PEG-Rh2 was analyzed with various concentrations of samples for 48 h by 3-(4,5-dimethylthiazol-2-yl)-2,5-diphenyl-tertazolium bromide (MTT) assay. After the incubation period, 10 μL of MTT solution (5 mg/mL) was added to each well. Plates were incubated for an additional 3–4 h, and the formed formazan was dissolved in DMSO. The absorbance of each well was recorded on a Synergy 2 multimode microplate reader at 570 nm (BioteK, Winooski, VT, USA). Untreated cells were used as a control (100%).

### 3.7. Measurement of Nitrite Levels

RAW 264.7 cells were pretreated with PEG-COOH, ginsenoside Rh1, ginsenoside Rh2, PEG-Rh1, or PEG-Rh2 for 1 h and then stimulated with 1 μg/μL lipopolysaccharide (LPS) in the presence of the samples and incubated for 48 h. The nitrite level in the medium was calculated using Griess reagent: 100 μL of supernatant was mixed with an equal volume of Griess reagent and measured at 540 nm by a microplate reader (Bio-Tek Instruments, Inc., Vinooski, VT, USA).

## 4. Conclusions

Korean ginseng is one of the most widely used remedies in Korea. It has unique triterpenoid saponins called ginsenosides, which are considered the active compounds responsible for the pharmacological effects of ginseng. The low solubility and tumor-targeted delivery of ginsenosides, which is due to avoiding off-target cytotoxicity, have been big challenges for clinical trials. To overcome these issues, water-soluble polymers with good biocompatible and biodegradable properties were loaded or conjugated with ginsenosides. Here, we described PEG conjugation applied to the ginsenosides Rh1 and Rh2 to increase solubility and cytotoxicity for tumor cells and inflammation. Finally, the anti-inflammation properties of PEG-Rh1 and PEG-Rh2 were evaluated through the inhibition of nitric oxide (NO) production.The higher drug conjugation efficiency, more solubility, smaller particle size with increased cytotoxicity to lung cancer of the PEG-Rh1 indicated that it exerts the overall efficacy of Rh1 for anticancer studies. However, reduced cytotoxicity of PEG-Rh2 than Rh2 with inhibition of NO production, the PEG-Rh2could be a potential drug candidate for inflammation related further studies.
